# Construction of a critical nitrogen dilution curve for maize in Southwest China

**DOI:** 10.1038/s41598-020-70065-3

**Published:** 2020-08-04

**Authors:** Lunjing Du, Qiang Li, Lan Li, Yawei Wu, Fang Zhou, Binxiang Liu, Bo Zhao, Xiaolong Li, Qinlin Liu, Fanlei Kong, Jichao Yuan

**Affiliations:** 1 Department of Crop Cultivation and Farming System, College of Agronomy, Sichuan Agricultural University, Chengdu, 611130 China; 2 College of Landscape Architecture and Life Science/Institute of Special Plants, Chongqing University of Arts and Sciences, Yongchuan, Chongqing, 402160 China; 3 Crop Institute of Chengdu Academy of Agricultural and Forestry Science, Chengdu, 611130 China

**Keywords:** Fertilization, Plant ecology

## Abstract

There is an urgent need for suitable nitrogen nutrition models for Southwest China, which take into account nutritional differences at the cultivar level, to provide scientific guidance for cultivar-specific fertilizer applications during maize production. In this study, the nitrogen-efficient maize cultivar Zhenghong 311 and the nitrogen-inefficient maize cultivar Xianyu 508 were used in a three-year field experiment and a 2-year field pot experiment with nitrogen application rates ranging from 0 to 450 kg·hm^−2^ to construct a critical nitrogen dilution curve model for each maize cultivar. The usefulness of this model to diagnose nitrogen status and evaluate maize fertilization needs was subsequently analyzed. We found that the critical nitrogen concentration in maize aboveground tissues was a power function of the biomass, described by the equations N_c_ = 26.126 W^−0.292^ and N_c_ = 25.826 W^−0.302^ for ZH 311 and XY 508 cultivars, respectively. The fitting degree of these equations was significant or highly significant, demonstrating the suitability of these models to diagnose N deficiency and fertilization needs in maize plants grown in the hilly areas of central Sichuan. A very significant linear positive correlation between the nitrogen nutrient index (NNI) and nitrogen concentration in the aboveground tissues was detected. Based on this, we calculated the nitrogen concentration (Nt) for an NNI equal to 1 at different maize growth stages in both cultivars and observed that the Nt value can be used as a reference index for nitrogen nutrition diagnosis. Additionally, we found a highly significant quadratic convex function relationship between the NNI (*y*) and the nitrogen fertilizer level (*x*). The following regression equations were derived for these maize cultivars with the data obtained from each growth period along five consecutive years (2011–2015): *y*_ZH 311_ = − 0.000005*x*^2^ + 0.003074*x* + 0.553206 (*R*^*2*^ = 0.5432**) and *y*_XY 508_ = − 0.000004*x*^2^ + 0.002914*x* + 0.512555 (*R*^*2*^ = 0.6279**). For an NNI value equal to 1, the nitrogen application level required was 224.07 kg·hm^−2^ for ZH 311 and 283.01 kg·hm^−2^ for XY 508, indicating that the suitable application rate for the nitrogen-efficient cultivar is lower than that for the nitrogen-inefficient cultivar. Our experimental data reinforce the concept that selecting nitrogen-efficient maize cultivars is an effective technical measure to reduce nitrogen input needs and increase nitrogen use efficiency during maize production.

## Introduction

Nitrogen is a nutrient required in large quantities for the growth and development of maize, and the primary limiting factor of crop growth and yield^1–4^. Increased nitrogen application is the most direct and effective way to enhance maize yield^5,6^. However, excessive nitrogen doses cause not only a significant decrease in grain yield and quality, but also a massive loss of nitrogen fertilizers, which results in increased production cost and environmental pollution^7,8^. Increasing maize grain yield without increasing nitrogen application rates is necessary to cope with the food crisis, maintaining environmental safety^9^. Under this scenario, the selection of nitrogen-efficient maize cultivars becomes an effective option to improve nitrogen use efficiency and prevent excessive nitrogen additions^10,11^.

The minimum nitrogen concentration necessary to achieve maximum biomass is known as a critical nitrogen concentration. The determination of this parameter in various key growth stages of maize is fundamental to optimize nitrogen application^12,13^. As plant biomass increases, the nitrogen concentration decreases (becomes diluted),this dilution phenomenon has been characterized as a power function^14^. Nitrogen uptake, nitrogen nutrition index (NNI), and cumulative nitrogen-deficit models based on the critical nitrogen concentration are effective methods to evaluate nitrogen status in crops^15,16^. The construction of critical nitrogen dilution curves for maize has been previously reported by several researchers. Plènet et al.^17^ established the following dilution curve to link the critical nitrogen concentration of maize (N_c_) and the dry weight of the aboveground biomass (W): N_c_ = 34.0 W^−0.37^. This model was applicable to maize crop development between emergence and 25 days after silking, and for W > 1 t·hm^−2^. Liang et al.^18^ and Li et al.^19^ established critical nitrogen dilution curves for the North China Region (N_c_ = 34.914 W^−0.4134^), and for the Guanzhong irrigation area (N_c_ = 25.3 W^−0.26^) and the Weibei Plain (N_c_ = 22.5 W^−0.27^), respectively. They asserted that these models enabled an accurate diagnosis of nitrogen status in maize plants grown in these regions. Although models applicable to both C_3_ and C_4_ crops have been developed, several studies demonstrated that substantial differences among the parameters included in the N_c_-W models exist due to differences in regions, crops, and cultivars^14,19^. Therefore, it is essential to assess the applicability of these models to particular agricultural settings.

The hilly region of Sichuan is one of the main maize-producing areas of China; however, only a few studies have analyzed the adequacy of the models mentioned for the diagnosis of nitrogen status in maize plants grown in this region^13,16^. Excessive nitrogen fertilization to achieve high maize yield is common in this region due to the relatively low soil fertility^5,6^. Therefore, there is an urgent need for suitable nitrogen nutrition models for this maize-producing area, which take into account nutritional differences at the cultivar level, to provide scientific guidance for cultivar-specific fertilizer applications during maize production. In the present study, two maize cultivars with different nitrogen use efficiencies were used as experimental materials in multi-year field experiments with different nitrogen application rates. Furthermore, critical nitrogen dilution curves for each cultivar were constructed and validated, to provide a theoretical guidance for nitrogen status evaluation, in order to generate reasonable recommendations regarding nitrogen fertilization during maize production in this region.

## Materials and methods

### Experimental materials

The nitrogen-efficient maize cultivar Zhenghong 311 and the nitrogen-inefficient maize cultivar Xianyu 508 (abbreviated as ZH 311 and XY 508, respectively), screened in previous studies^20–23^, were used as experimental materials in the present study. Both cultivars have similar growth periods of approximately 120 d.

### Study area

**Figure 1:**
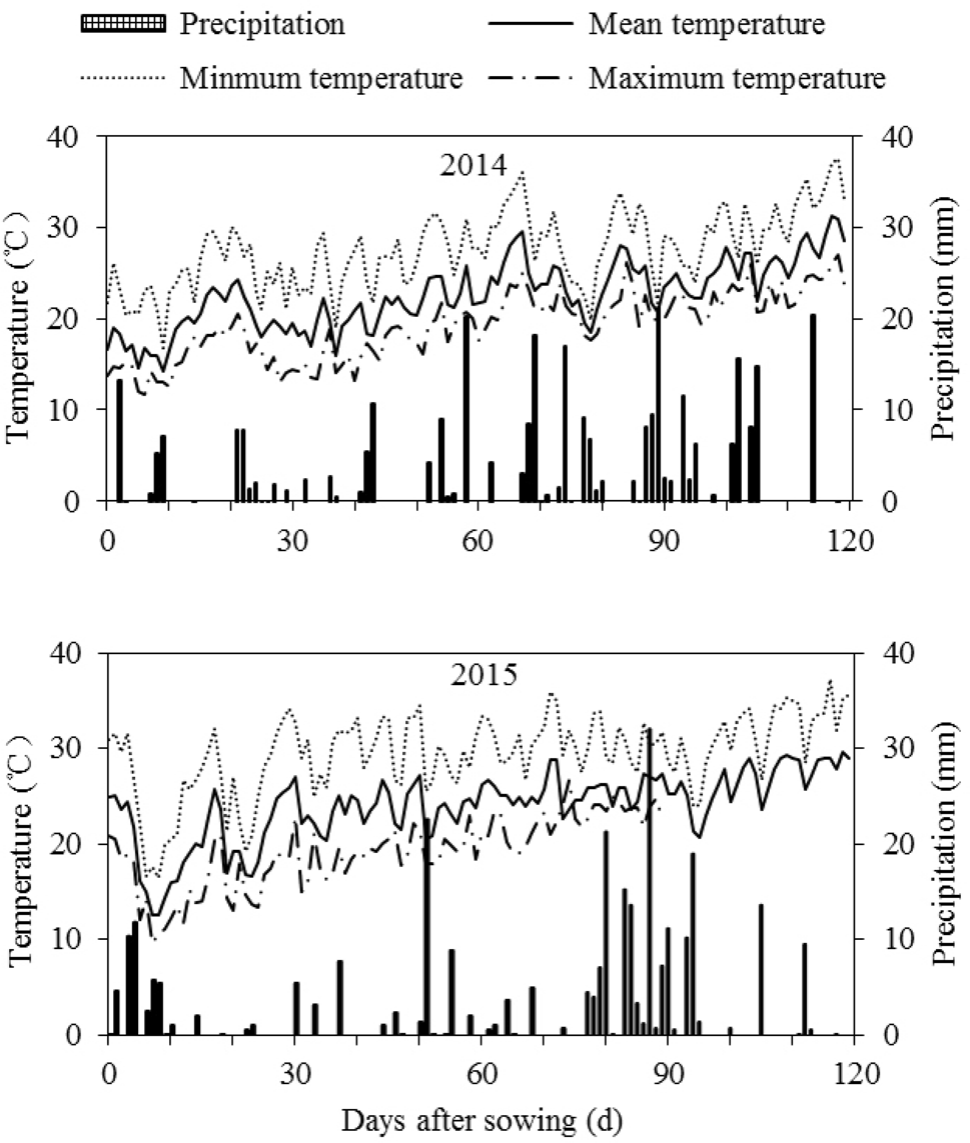
Meteorological data during spring maize growth period in Sichuan (2014–2015).

Two experiments were conducted from 2011 to 2015 in an experimental site close to Jianyang City, Sichuan Province of China (30°04ʹ–30°39′N, 104°11′–104°53′E). The first one was conducted from 2011 to 2013 and was a field experiment; the second one was from 2014 to 2015 and was a field pot experiment. Jianyang City has a subtropical monsoon climate, with an average annual rainfall of 874 mm, average annual temperature of 17 °C, and annual frost-free period of 311 d. Figure 1 and Table 1 show the meteorological data during the maize growth period and soil basic fertility in our field pot experiments, 2014–2015, respectively.

**Table 1:** Soil basic fertility in 2014 and 2015.

Years	2011–2013	2014	2015
Organic matter (g·kg^−1^)	16.60	15.75	15.30
Total N (g·kg^−1^)	1.24	1.75	1.56
Total P (g·kg^−1^)	0.73	0.57	0.40
Total K (g·kg^−1^)	12.54	12.61	8.25
Alkali hydrolysable N (mg·kg^−1^)	25.22	39.26	36.34
Olsen-P (mg·kg^−1^)	13.54	2.55	2.27
Exchangeable K (mg·kg^−1^)	138.75	139.33	128.50
pH	8.63	7.59	8.16

### Experimental design

In 2011–2013, a fixed location field experiment was conducted using wheat/maize/sweet potato crop rotation. A split-plot experimental design was adopted, with the main-plot factor being the maize cultivar (two levels: ZH 311 and XY 508) and the sub-plot factor being the nitrogen fertilizer level (six levels: 0, 90, 180, 270, 360, and 450 kg·hm^−2^). The experiment was repeated thrice with 36 sub-plots each having an area of 20 m^2^. Direct seeding was performed in late March with a plant spacing of (1.5 m + 0.5 m) × 0.2 m, and crops were grown in monoculture with a planting density of 50,000 plants·hm^−2^. Nitrogen fertilizer was applied using a basal fertilizer, seedling fertilizer, and panicle fertilizer at a ratio of 4:1:5; calcium superphosphate and potassium chloride were applied as basal fertilizers at rates of 600 and 150 kg·hm^−2^, respectively. Other plant management practices were implemented in accordance with local requirements for high crop yields.

In 2014 and 2015, field pot experiments were conducted, and soil samples were collected to carry out the analysis of soil nitrogen balance. A two-factor randomized block design was adopted with factor A being the maize cultivar (ZH 311 and XY 508) and factor B being the nitrogen fertilizer level. Three nitrogen fertilizer levels, N0 (0 kg·hm^−2^), N150 (150 kg·hm^−2^), and N300 (300 kg·hm^−2^), were set in 2014, and 4 nitrogen fertilizer levels, N0 (0 kg·hm^−2^), N150 (150 kg·hm^−2^), N300 (300 kg·hm^−2^), and N450 (450 kg·hm^−2^), were set in 2015. The size of the pots used was 0.45 cm (height) × 0.3 cm (diameter). The experimental soil was obtained from the 0–30 cm soil layer of a long-term maize cultivation area and air-dried. Organic debris such as roots was removed from the soil, which was subsequently sieved through a 0.5-cm-mesh screen before use. Each pot was filled with 20 kg of the experimental soil in the following manner: 18 kg of soil was placed in the pot, basal fertilizer was applied, and the remaining soil (2 kg) was placed on the surface. Seedlings were cultivated in late March and transferred to the pots during the two-leaf stage, with two seedlings planted per pot. The pots were buried in pre-dug ditches at a depth of 0.3 m and placed in alternating wide and narrow rows (1.4 m + 0.4 m) with a planting density of 52 500 plants·hm^−2^.

Nitrogen was applied following the experimental scheme of 50% as basal fertilizer and 50% as panicle fertilizer; calcium superphosphate and potassium chloride were used as basal fertilizers at rates of 600 kg·hm^−2^ and 150 kg·hm^−2^, respectively. Other plant management practices were implemented in accordance with local production requirements. Treatments were tested in triplicate; 20 pots per treatment and replicate were included.

### Plant measurements and nitrogen nutrition assessment

#### Dry matter accumulation

During the silking and maturity stages of maize plants grown in 2011–2013, and the jointing, bell-mouth (V12), silking, grain-filling, and maturity stages of maize plants grown in 2014–2015, four representative plants displaying similar growth were selected, fixed at 105 °C for 30 min, dried to a constant weight at 80 °C, and weighed.

#### Nitrogen concentration and nitrogen accumulation

Once weighed, dried samples were pulverized and sieved through a 60-mesh screen. The Kjeldahl method was used to determine total nitrogen content (nitrogen concentration), which was subsequently used for calculating nitrogen accumulation.

#### Construction and validation of critical nitrogen dilution curves

The dry matter accumulation and nitrogen accumulation data obtained from the field experiment carried out in 2011–2012 and the field pot experiment performed in 2015 were used for the construction of the nitrogen concentration dilution curves. The experimental data from the field experiment in 2013 and the field pot experiment in 2014 were used for validation. The critical nitrogen dilution models of the present study were established based on the critical nitrogen concentration theory proposed by Justes et al*.*^19^ and adopting the method proposed by Herrmann et al.^24^. According to these authors, plant biomass initially increases with increasing nitrogen content, but subsequently reaches a plateau and no longer increases with further nitrogen content increase. Based on this, the data obtained for different treatments during the same period were grouped to develop a segmented function, which can describe the relationship between biomass and nitrogen content, as follows:

1 $$ W = \left\{ {\begin{array}{*{20}l}    {a \cdot Na^{2}  + b \cdot Na + c} \hfill & {Na \le Nc(quadraticpart)} \hfill  \\    m \hfill & {Na \ge Nc(plateaupart)} \hfill  \\   \end{array} } \right. $$

where, W is the aboveground biomass (t·hm^−2^); *a*, *b,* and *c* are parameters; m is the average aboveground dry matter when the mass no longer changes significantly; N_a_ is the actual nitrogen concentration in the aboveground biomass (g·kg^−1^); and N_c_ is the critical nitrogen concentration (g·kg^−1^).

The critical nitrogen dilution model is based on the following equation:

2 $$ {\text{N}}_{{\text{c}}} = a\cdot{\text{W}}^{ - b} $$

where *a* is the critical nitrogen concentration in plants when the dry weight of the aboveground biomass is 1 t·hm^−2^, and *b* is the dilution coefficient, a statistical parameter associated with the slope of the curve.

To validate the critical nitrogen curves constructed, the NNI was calculated as follows:

3 $$ {\text{NNI }} = {\text{ N}}_{{\text{a}}} /{\text{N}}_{{\text{c}}} $$

and interpreted based on previous reports, according to which NNI < 1 indicates inadequate nitrogen nutrition, NNI = 1 indicates appropriate nitrogen nutrition, and NNI > 1 indicates excessive nitrogen nutrition^25,26^.

### Data processing

Experimental data were organized using Microsoft Excel 2010, and the analysis of variance was performed using SPSS 19.0 software. The least significant difference test was employed to evaluate the significance of differences between means.

## Results

### Effect of nitrogen application rate on aboveground dry matter accumulation

Maize aboveground dry matter increased continuously with the progression of the growth cycle during 2014 and 2015, reaching a maximum in the maturity stage (Table 2). The effect of nitrogen application rate, maize cultivar, and their interaction on dry matter accumulation in maize growth stages was significant. Dry matter accumulation in the nitrogen-efficient cultivar ZH 311 during all the growth stages analyzed was significantly higher than that in the nitrogen-inefficient cultivar XY 508. In 2014, dry matter accumulation in ZH 311 during the jointing, bell-mouth, silking, grain-filling, and maturity stages was 20.49%, 25.15%, 28.04%, 24.89%, and 41.39% higher than that measured in the cultivar XY 508, respectively; the corresponding differences in 2015 were 6.67%, 23.01%, 30.33%, 22.33%, and 27.75%. It may be observed that nitrogen fertilization significantly promoted dry matter accumulation in all growth stages, and differences existed in these promoting effects between different years, indicating that soil fertility and meteorological conditions are also involved in the effect of nitrogen application on dry matter accumulation.

**Table 2:** Effect of nitrogen application rate on dry matter accumulation in spring maize (t·hm^-2^).

Cultivar	Nitrogen	Jointing stage		Bell-mouth stage		Silking stage		Filling stage		Maturity stage	
		2014	2015	2014	2015	2014	2015	2014	2015	2014	2015
ZH 311	N0	0.33 d	0.19 e	0.95 d	0.77 f	1.71 d	1.42 f	2.66 d	2.31 e	4.40 e	2.67 f
	N150	0.70 b	0.82 bc	2.76 b	2.45 c	4.73 b	4.83 d	7.55 b	8.12 c	12.73 b	10.72 c
	N 300	0.80 a	0.95 a	3.41 a	3.00 a	5.58 a	6.02 a	8.69 a	9.85 a	14.19 a	12.09 a
	N 450	–	0.81 c	–	2.66 b	–	5.68 b	–	9.17 b	–	11.43 b
	**Av**	**0.61 A**	**0.69 A**	**2.37 A**	**2.22 A**	**4.01 A**	**4.49 A**	**6.30 A**	**7.36 A**	**10.44 A**	**9.22 A**
XY 508	N 0	0.29 d	0.17 e	0.80 d	0.59 g	1.21 e	0.80 g	1.90 e	1.65 f	3.17 f	1.85 g
	N 150	0.58 c	0.76 d	2.36 c	2.01 e	4.13 c	4.31 e	6.81 c	6.89 d	8.64 d	8.13 e
	N 300	0.63 c	0.81 b	2.44 c	2.41 cd	4.35 bc	5.00 c	7.02 c	8.31 c	10.28 c	10.28 cd
	N 450	–	0.78 c	–	2.31 d	–	5.10 c	–	8.12 c	–	9.75 d
	**Av**	**0.50 B**	**0.63 B**	**1.87 B**	**1.83 B**	**3.23 B**	**3.80 B**	**5.24 B**	**6.24 B**	**7.36 B**	**7.50 B**
F value	Cultivar	44.46**	49.44**	81.68**	173.67**	46.43**	797.95**	74.42**	535.47**	279.23**	125.68**
	Nitrogen	230.78**	2,677.77**	518.58**	965.93**	365.81**	7,318.08**	820.33**	4,595.27**	795.37**	723.05**
	Cultivar × Nitrogen	5.24*	14.24**	19.06**	8.04**	4.06*	22.36**	6.15*	14.67**	25.17**	5.59**

Different lowercase and uppercase letters denote significant differences at 0.05 and 0.01 levels, respectively; * and ** indicate significant difference or correlation at 0.05 and 0.01 levels, respectively.

Differences in total dry matter accumulation between the cultivars were relatively large when nitrogen was not added (N0), or a low-nitrogen dose (N150) was applied. However, these differences gradually decreased as the nitrogen application rate increased (N300 and N450). In particular, total dry matter accumulation in ZH 311 in 2015 subjected to N0, N150, N300, and N450 treatments was higher than that in XY 508 by 44.32%, 31.86%, 17.61%, and 17.23%, respectively. This shows that the advantage of the nitrogen-efficient cultivar over the nitrogen-inefficient cultivar in dry matter accumulation was mainly manifested under low nitrogen fertilizer levels.

### Effect of nitrogen application rate on aboveground nitrogen accumulation

As shown in Table 3, aboveground nitrogen accumulation in maize gradually increased with the progression of the growth cycle, reaching a maximum in the maturity stage. The effect of cultivar, nitrogen application rate, and their interaction on nitrogen accumulation throughout maize growth stages was significant. ZH 311 accumulated more nitrogen during the different growth stages in 2014 and 2015 than XY 508. In 2014, nitrogen accumulation in ZH 311 during the jointing, bell-mouth, silking, grain-filling, and maturity stages was 16.09%, 24.52%, 21.67%, 19.98%, and 28.16% higher than that in XY 508, respectively; the corresponding differences between cultivars in 2015 were 11.32%, 22.24%, 18.65%, 19.68%, and 20.96%. In that year, nitrogen accumulation in ZH 311 increased in all growth stages until the nitrogen application rate increased, with maximum nitrogen accumulation achieved with the N300 treatment in all growth stages. However, in 2015, nitrogen accumulation in ZH 311 exhibited an initial increase and a subsequent decrease as nitrogen application rates increased, with maximum nitrogen accumulation under the N300 treatment. In the same year, nitrogen accumulation increased in XY 508 with increasing nitrogen doses during all maize growth stages except for the jointing stage. Maximum nitrogen accumulation was achieved in XY 508 under the N300 treatment in 2014 and N450 treatment in 2015.

**Table 3:** Effect of nitrogen application rate on nitrogen accumulation in spring maize (kg·hm^−2^).

Cultivar	Nitrogen	Jointing stage		Bell-mouth stage		Silking stage		Filling stage		Maturity stage	
		2014	2015	2014	2015	2014	2015	2014	2015	2014	2015
ZH 311	N 0	4.51d	2.27e	8.10 e	10.02f.	19.02 d	14.07 g	24.30e	19.89 g	32.79d	21.96 g
	N150	23.60 b	26.20 c	47.06c	31.96d	72.32 c	66.60e	90.36c	86.51e	108.92b	100.00e
	N 300	27.75a	32.10 a	70.54a	56.45a	116.86 a	100.64a	119.74a	119.17a	140.27a	133.01a
	N 450	–	27.18b	–	46.68b	–	92.76b	–	114.76b	–	127.34b
	Av	**18.62A**	**21.93A**	**41.90 A**	**36.28 A**	**69.40 A**	**68.52 A**	**78.13 A**	**85.08 A**	**93.99 A**	**95.58 A**
XY 508	N 0	4.16d	2.66e	5.89f.	7.00 g	11.78 e	7.90 h	15.36f.	13.11 h	21.00e	15.15 h
	N 150	20.60 c	22.79d	43.91d	24.66e	67.27 c	55.04f.	81.94d	70.33f.	86.96c	75.68f.
	N 300	23.35b	27.36b	51.16b	41.33c	92.06 b	79.87d	98.07b	96.48d	112.05b	106.65d
	N 450	–	25.98c	–	45.71b	–	88.17c	–	104.42c	–	118.60c
	Av	**16.04 B**	**19.70B**	**33.65 B**	**29.68 B**	**57.04 B**	**57.75 B**	**65.12 B**	**71.09 B**	**73.34 B**	**79.02 B**
F value	Cultivar (C)	153.89**	417.81**	302.12**	473.21*	84.43**	739.70**	150.09**	276.71**	508.66**	276.85**
	Nitrogen (N)	3,981.19**	12,871.84**	4,553.85**	3,796.08**	1,484.18**	8,935.41**	2,530.53**	2,669.12**	4,159.34**	2,375.17**
	C × N	32.53**	108.68**	137.86**	106.44**	21.58**	84.86**	16.64**	17.19**	27.33**	26.29**

Different lowercase and uppercase letters denote significant differences at 0.05 and 0.01 levels, respectively; * and ** indicate significant difference or correlation at 0.05 and 0.01 levels, respectively.

### Construction and validation of critical nitrogen dilution curves

**Figure 2:**
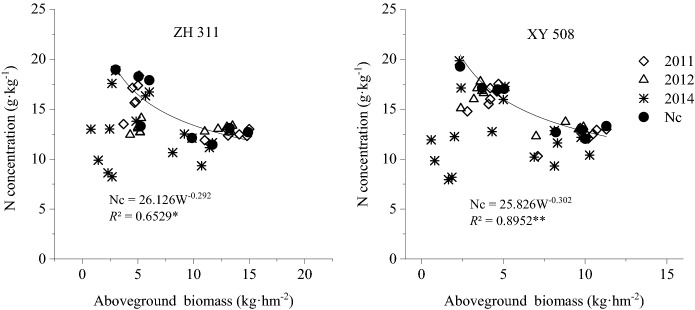
Critical nitrogen concentration dilution curve for spring maize in Sichuan.

In the present study, the relationship between nitrogen concentration and dry matter accumulation during the silking and maturity stages in 2011 and 2012 and during the bell-mouth, silking, grain-filling, and maturity stages in 2015 were used to determine critical nitrogen concentrations and maximum dry matter accumulations (the jointing stage was excluded because dry matter accumulation < 1 t·hm^−2^) (Fig. 2). Subsequently, the following critical nitrogen concentration dilution curve equations were derived: N_c_ = 26.126 W^−0.292^ for ZH 311 and N_c_ = 25.826 W^−0.302^ for XY 508. The goodness-of-fit of these curves was found to be significant or highly significant, making them suitable for the diagnosis of the nitrogen status of maize grown in the hilly areas of Sichuan.

**Figure 3:**
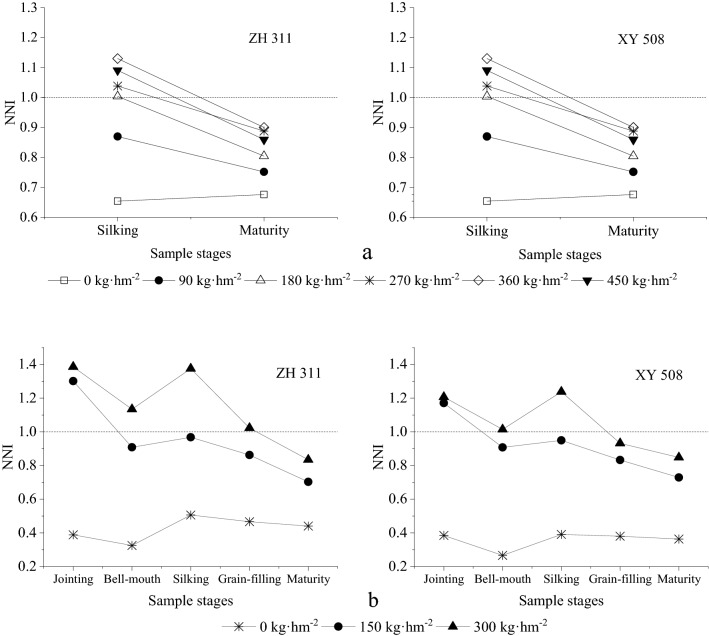
Nitrogen nutrition index (NNI) of spring maize subjected to different N treatments in Sichuan. (**a**: 2013; **b**: 2014).

The NNI provides an indication of the nitrogen status of maize. An NNI value of 1 or higher indicates that maize growth is not limited by nitrogen supply, and an NNI value below 1 indicates that maize growth is limited by nitrogen deficiency. As shown in Fig. 3 the nitrogen fertilizer level significantly affected maize NNI. In 2013, the NNI exhibited an initial increase and a subsequent decrease as nitrogen application rates increased, achieving its maximum value with the N360 treatment. In 2014, the NNI increased with increasing nitrogen application rates, with the extent of this rise decreasing gradually; the maximum NNI was observed under the N300 treatment (Fig. 3).

Subsequently, the NNI in different growth stages was compared. Although the jointing stage is considered more sensitive to nitrogen application, the smaller plant size led to an inferior nitrogen requirement. Therefore, the NNI during the jointing stage exceeded 1 at a nitrogen fertilizer level of 150 kg·hm^−2^. With the progression of the growth cycle, dry matter accumulation increased continuously, whereas nitrogen concentration decreased continuously, resulting in higher nitrogen requirements. Consequently, the NNI during the maturity stage was less than 1, even under a nitrogen fertilizer dose of 300 kg·hm^−2^, demonstrating substantial differences in nitrogen demands among different growth stages.

### Adequate nitrogen application levels

**Figure 4:**
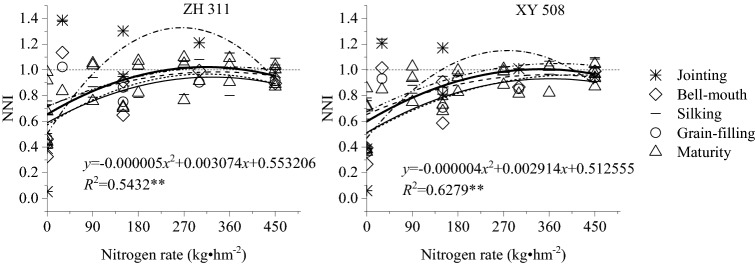
Relationship between NNI and nitrogen application in spring maize in Sichuan, 2011–2015.

Figure 4 shows that the NNI among maize growth stages in both cultivars exhibited an initial increase and a subsequent decrease as the nitrogen application rate increased. The regression analysis performed on data obtained for various maize growth stages over a 5-year period indicated that the NNI (*y*) and nitrogen fertilizer level (*x*) were related by a convex quadratic function, with the regression equations for ZH 311 and XY 508 being *y*_ZH 311_ = -0.000005*x*^2^ + 0.003074x + 0.553206 (*R*^2^ = 0.5432^**^) and *y*_XY 508_ = -0.000004*x*^2^ + 0.002914*x* + 0.512555 (*R*^2^ = 0.6279^**^), respectively.

When the NNI = 1, the nitrogen application rates for ZH 311 (Nf_ZH 311_) and XY 508 (Nf_XY 508_) were 224.07 and 283.01 kg·hm^−2^, respectively. It may be observed that the Nf value of XY 508 was higher than that of ZH 311, indicating that the nitrogen-efficient cultivar had a lower nitrogen requirement than the nitrogen-inefficient cultivar. This finding suggests that nitrogen application rates in maize nitrogen-efficient cultivars might be reduced compared with those in nitrogen-inefficient cultivars.

### Adequate nitrogen concentration levels

The correlation analysis demonstrated the existence of a highly significant positive correlation between the NNI and plant nitrogen concentration in maize during all growth stages. Table 4 shows the regression equation and the calculated plant nitrogen concentration (Nt) for NNI = 1, based on this equation. When the actual aboveground nitrogen concentration is lower than the Nt at a given growth stage (NNI < 1), it may be considered that nitrogen deficiency exists. If the actual aboveground nitrogen concentration is higher than the Nt value, plants are exposed to excess nitrogen. Therefore, plant nitrogen status can be diagnosed based on the actual nitrogen concentration and Nt values.

**Table 4:** Relationship between NNI and Na in spring maize grown in Sichuan, 2011–2015.

Cultivar	Stages	Equation parameters				Nt (NNI = 1) g·kg^−1^
		a	b	*R*^2^	P	
ZH 311	Jointing stage	1.8593	26.3535	0.9896	0.0001	28.2128
	Bell-mouth stage	4.9209	13.9771	0.9597	0.0001	18.8980
	Silking stage	3.6424	12.2915	0.9193	0.0001	15.9339
	Filling stage	4.9811	8.3991	0.9502	0.0002	13.3802
	Maturity stage	2.7534	9.3924	0.8884	0.0001	12.1458
XY 508	Jointing stage	1.6204	27.4420	0.9784	0.0001	29.0624
	Bell-mouth stage	4.0430	15.5895	0.9636	0.0001	19.6325
	Silking stage	4.7279	11.7615	0.8686	0.0001	16.4894
	Filling stage	4.6722	8.6847	0.9652	0.0001	13.3569
	Maturity stage	3.1772	9.3720	0.8762	0.0001	12.5492

Table 4 also shows that plant nitrogen concentrations decreased with the progression of the maize growth cycle. The nitrogen concentration in ZH 311 remained lower than that in XY 508 throughout the experiment; this finding can be attributed to differences in the nitrogen uptake, utilization, and transformation abilities between these two cultivars.

## Discussion

### Applicability of critical nitrogen dilution curves for spring maize cultivated in the hilly areas of central Sichuan

Critical nitrogen dilution curves are commonly used for the diagnosis of plant nitrogen status and determination of optimum nitrogen fertilization schemes and have become a key method for the assessment of crop nitrogen status in China and other countries^26^. Several studies have shown that parameters *a* and *b* depend on variables such as cultivar, soil fertility, and meteorological conditions^27–30^.

In general, higher nitrogen uptake and plant nitrogen concentrations occur in fertile soils, resulting in higher *a* and *b* values. On the contrary, nitrogen-efficient cultivars have a stronger ability to take up nitrogen, leading to higher *a* values^28,31–33^. The parameters of the critical nitrogen dilution models established in the present study for two maize cultivars (ZH 311: N_c_ = 26.126 W^−0.292^, XY 508: N_c_ = 25.826 W^−0.302^) are similar to those proposed by Li et al.^13^ for summer maize in the Guanzhong irrigation area (N_c_ = 22.5 W^−0.27^) and spring maize in the Weibei Plain (N_c_ = 25.3 W^−0.26^). However, they were significantly different from those included in the critical nitrogen dilution model established by Liang et al.^18^ for summer maize in the North China Plain (N_c_ = 34.914 W^−0.4134^). This may be attributed to differences in soil fertility conditions and cultivars used. In this sense, it is worth mentioning that basic fertility in our experimental field was higher than that in the Guanzhong irrigation area and Weibei Plain, but lower than that in the North China Plain, and the magnitudes of *a* and *b* followed the same order. A higher *a* value was reported by Liang et al*.*^18^ due to the utilization of the nitrogen-efficient cultivar Zhengdan 958 in the North China Plain, characterized by higher soil fertility. Li et al*.*^13^ also used in their experiments the cultivar Zhengdan 958 in the Weibei Plain and reported a higher *a* value than that obtained with the cultivars Nongda 108, Shendan 10, and Qinlong 11 in the Guanzhong irrigation area. In the present study, the *a* value of ZH 311 was also slightly higher than that of XY 508, indicating a stronger ability of ZH 311 to take up nitrogen than that of XY 508 and further validating the high nitrogen efficiency of the former cultivar^20,22^. In addition, this result also shows that the models for critical nitrogen dilution curves established and validated using data from field experiments conducted over five consecutive years are adequate and may be used for the tested cultivars ZH 311 and XY 508.

Parameter *b* in the critical nitrogen dilution curves represents the dilution coefficient. Ma et al.^26^ reported that the magnitude of *b* is dependent on the ratio of nitrogen concentration to dry matter, with higher ratios resulting in higher *b* values. According to Yang et al.^34^^,^ lower *b* values indicate that plants can retard the nitrogen concentration decrease that occurs until biomass grows, thereby enabling more stable and sustained plant growth. In the present study, the *b* value of ZH 311 was slightly lower than that of XY 508, indicating that ZH 311 had steadier growth, was less influenced by external nitrogen levels, and achieved more stable dry matter production and yields than XY 508 under the same soil fertility conditions.

### Diagnosis of nitrogen status and cultivar-specific fertilizer application based on the critical nitrogen dilution models

Plant nutrient status reflects the amount of nutrient supply from the soil, plant nutrient requirements, and plant’s ability to take up and utilize nutrients. The NNI, which may be deduced from critical nitrogen dilution models, not only allows diagnosing nitrogen status but also enables the quantification of nitrogen stress in crops^26,34,35^. Higher NNI values indicate higher nitrogen nutrition levels. The NNI of < 1 corresponds to nitrogen inadequacy or deficiency situations, which require appropriate nitrogen supplementation using fertilizers. The NNI of > 1, indicates excess nitrogen in plants, which should be rectified through the control of nitrogen applications^14–19^.

Our correlation and regression analyses indicated the existence of a highly significant positive correlation between the NNI and nitrogen concentration in maize plants and a convex quadratic function between the NNI and nitrogen fertilizer level. Based on the regression equation, the nitrogen concentration Nt and the nitrogen fertilizer level Nf when the NNI = 1 can be calculated and used as reference indices to assess maize nitrogen status and fertilization needs. Table 4 shows the Nt values calculated from data obtained in field experiments conducted over five consecutive years in the hilly areas of central Sichuan. As maize growth progressed, the Nt values of both cultivars gradually decreased, with the Nt values of ZH 311 lower than those of XY 508 during all growth stages. The Nf values calculated from data of all growth stages were 224.07 and 283.01 kg·hm^−2^ for ZH 311 and XY 508, respectively. The significantly lower Nf value of ZH 311 than that of XY 508 indicates that the formulation of cultivar-specific fertilization guidelines, selection of nitrogen-efficient cultivars, and implementation of effective technical measures to enhance nitrogen use efficiency are key approaches to achieve China’s target of zero growth in the use of chemical fertilizers by 2020.

## Conclusions

The critical nitrogen concentration in maize aboveground tissues was a power function of the biomass, described by the equations N_c_ = 26.126 W^−0.292^ and N_c_ = 25.826 W^−0.302^ for ZH 311 and XY 508, respectively. The fitting degree of these equations was significant or highly significant, demonstrating the suitability of these models to diagnose N deficiency and fertilization needs in maize plants grown in the hilly areas of central Sichuan. A very significant linear positive correlation between the NNI and nitrogen concentration in the aboveground tissues was detected. Based on this, we calculated the nitrogen concentration (Nt) for an NNI equal to 1 at different maize growth stages in both cultivars and observed that the Nt value can be used as a reference index for nitrogen nutrition diagnosis. Additionally, we found a highly significant quadratic convex function relationship between the NNI (*y*) and the nitrogen fertilizer level (*x*). The following regression equations were calculated for these maize cultivars with the data obtained from each growth period along five consecutive years (2011–2015): *y*_ZH 311_ = -0.000005*x*^2^ + 0.003074*x* + 0.553206 (*R*^2^ = 0.5432**) and *y*_XY 508_ = − 0.000004*x*^2^ + 0.002914*x* + 0.512555 (*R*^2^ = 0.6279**). For an NNI value equal to 1, the nitrogen application levels required were 224.07 kg·hm^−2^ for ZH 311 and 283.01 kg·hm^−2^ for XY 508, indicating that the suitable application rate in the nitrogen-efficient cultivar is lower than in the nitrogen-inefficient cultivar. Our experimental data reinforce the concept that selecting for nitrogen-efficient maize cultivars is an effective technical measure to reduce nitrogen input needs and increase nitrogen use efficiency during maize production.
